# Supplementary material for: Are carbon emissions trading and green financial instruments synergistic? -Comprehensive quantitative research based on content analysis
Source: PLoS One. 2024 Mar 7;19(3):e0298601. doi: 10.1371/journal.pone.0298601 (PMC10919601; doi:10.1371/journal.pone.0298601)
Supplement: S1 Data — (ZIP) [file pone.0298601.s001.zip › Data/地级市Z_F工作报告.zip!/░▓╗╒/┬φ░░╔╜╩╨/└┤╫╘╣1⁄2╓┌║┼í╛┬φ┐╦╩2╛▌═°í┐_2019.docx]

# **政府工作报告（2019）**

【字体大小：[大](http://zwgk.mas.gov.cn/openness/detail/content/javascript:doZoom(18)) [中](http://zwgk.mas.gov.cn/openness/detail/content/javascript:doZoom(15)) [小](http://zwgk.mas.gov.cn/openness/detail/content/javascript:doZoom(14))】

——2019年1月6日在马鞍山市第十六届人民代表大会第二次会议上

市长  左俊

各位代表：

    现在，我代表市人民政府，向大会报告政府工作，请予审议，并请市政协委员和其他列席人员提出意见。

****一、2018年工作回顾****

    2018年是我市发展不平凡的一年。宏观环境复杂多变，改革发展任务艰巨。在省委、省政府和市委的坚强领导下，全市人民深入学习习近平新时代中国特色社会主义思想特色社会主义思想，认真贯彻党的十九大精神，坚持稳中求进工作总基调，按照“三个走在前列”的要求，同心协力、克坚攻难、扎实苦干，较好地完成了市十六届人大一次会议确定的各项目标任务，在推动“生态福地、智造名城”建设上迈出了坚实步伐。

    ——经济运行稳中有进。预计实现地区生产总值1800亿元、增长8.5%，超过全省0.4个百分点；财政收入270.66亿元、增长10.3%；固定资产投资增长10%；规上工业增加值增长9%；社会消费品零售总额增长13%；进出口总额44亿美元、增长15.8%。GDP总量有望继续保持全省第3位，经济运行综合考核继续保持全省第一方阵。

    ——重大项目顺利推进。巢马城际铁路签署省市共建协议；马鞍山-南京-镇江城际铁路线获国家批复；宁马城际铁路、宁和城际铁路启动预可研编制；商合杭高铁含山段、郑蒲港铁路加快建设；宁马高速改造前期有序推进；长江马鞍山段二期整治顺利开工。奥克斯空调、华旺新材料等一批重大项目加速建设，国轩高科、华谊名品等一批重大项目建成投产。

    ——创新能力持续提升。三大战新基地建设取得新进展。战略性新兴产业产值增长19%，高新技术产业增加值增长17%。获批建设国家创新型城市，获评国家知识产权示范城市，城市创新竞争力居全国第67位；每万人发明专利19.8件，全社会研发经费支出占GDP比重2.55%，均居全省第3。

    ——生态环保成效明显。举全市之力，深入抓好中央、省环保督察交办问题整改，扎实推进水清岸绿产业优美丽长江（马鞍山）经济带建设，解决了一大批多年想解决而未得到解决的环保问题，生态环境质量再上新台阶。PM2.5、PM10平均浓度下降10%、8.4%，空气优良天数同比增加18天，空气质量居全省第4，创历史最好水平；长江干流5个国控断面水质达标率100%。获批全国城市黑臭水体治理示范城市。

    ——民生保障得到加强。城镇和农村居民人均可支配收入分别达44920元、21190元，增长8.5%和9.5%，两项收入继续保持全省第1；城镇登记失业率2.61%，低于全国全省平均水平；全面完成1551人年度脱贫任务；烟花爆竹禁放、犬只规范管理、共享单车治理等有效施行。人民群众获得感、幸福感、安全感进一步增强。

    一年来，我们主要做了以下工作：

****（一）打好三大攻坚战，着力补齐发展短板。****有效防控重大风险。持续开展互联网金融专项整治、非法交易场所清理整顿、非法集资排查处置专项行动，守住了不发生区域性、系统性金融风险的底线。开展政府债务“四清四实”专项整治，实行政府债务总量和成本“双控”，全市政府债务率下降1.1个百分点，政府债务和财政风险总体可控。扎实开展精准脱贫。深入实施脱贫攻坚“八大工程”“百日攻坚”专项行动。全省产业扶贫、“千企帮千村”现场会在我市召开，安徽联邦农业、满篮鲜电子商务获全国表彰。强力推进污染防治。全面展开蓝天、碧水、净土“三大保卫战”。建成区28条黑臭水体基本完成治理，5个集中式饮用水水源地水质达标率100%。单位GDP能耗下降6.2%，单位固投消耗新增建设用地量考评居全国第9、全省第1。当涂县国土资源节约利用工作获国务院表彰，市经开区获评国家级绿色园区。

****（二）支持实体经济，着力推动产业升级。****聚焦发展先进制造业。马钢轨道交通、迈特诺、津上机床、沈阳机床、海思达、沪宁机械等一批龙头企业进一步做大做强，新能源、电子信息、生物医药等战新产业取得新的发展。

    改造升级传统产业。实施技改项目350个，技改投资增长10%以上，引导企业兼并重组35家。加快“两化”融合步伐，新增省级智能工厂1家、数字化车间7个。国星生化智能工厂入选国家级智能制造试点示范项目，山鹰造纸荣登安徽民营企业前10强。马钢销售收入、利润、上交税金均实现大幅增长，创历史最好水平，为全市经济发展做出了重要贡献。加快发展现代服务业。第三产业增加值占GDP比重41.6%，比上年提升2.8个百分点。入选全国数字经济百强城市，百助网络迈进中国互联网企业百强，浙阿跨境电商产业园进入省级名录。国家级物流标准化试点项目基本完成。采石5A级景区创建有序推进。

****（三）强化项目支撑，着力扩大有效投入。****深入开展“双集中”活动。实施亿元以上重点项目750个，完成投资1110亿元。105个市级重点调度项目完成投资370亿元，543个省“大新专”项目完成投资910亿元。招大引强成效明显。实际利用外商直接投资24.38亿美元、增长7%，总量居全省第3；实际利用省外亿元以上项目到位资金935亿元、增长12%。冠辉新材料、纳百川汽车动力等17个20亿元以上重大项目签约落地。社会民生项目加快建设。市卫生应急处置指挥中心、档案资源管理中心顺利开工，市妇幼保健院综合楼、气象防灾减灾指挥中心加快推进，马鞍山幼师新校区、市心理健康中心、残疾人康复中心竣工运行。启动实施10大环保重点工程，慈湖水厂取水头部迁改工程基本完工，固体废物回收利用、污水处理厂提标改造等项目开工建设。

****（四）全面深化改革，着力激发内生动力。****持续推进供给侧结构性改革。马钢退出炼铁产能100万吨、炼钢产能128万吨，去产能“三年行动计划”全面完成，“三供一业”和幼教中心移交顺利实施；去库存与稳房价并重，房地产市场平稳健康发展；落实降本减负政策措施，减免各类税费45.57亿元；加大金融服务实体经济力度，中小微民营企业贷款余额186亿元，直接融资189亿元。统筹推进“放管服”改革。证照分离、减证便民、企业开办全程网上办、相对集中行政许可权等改革试点扎实推进。“互联网+政务服务”不断深化，“一网一门一次”改革基本完成，政务服务一张网实现四级全覆盖。深入推进“四送一服”双千工程，开展“四最”营商环境专项治理。协调推进重点领域改革。11个市级医联体、专科联盟、县域医共体成功组建，公立医院编制周转池制度初步建立。深化城市执法体制改革，住建领域行政处罚权统一行使。农村集体产权制度及“三变”改革整市推进。河长制、湖长制、林长制以及生态保护补偿机制、生态环境损害赔偿制度初步建立。开发园区优化整合全面完成，郑蒲港新区法定机构改革试点启动。

****（五）坚持双轮驱动，着力增强发展动能。****加强创新能力建设。培育科技“小巨人”企业87家、认定45家，高新技术企业突破400家，国家级农业科技园区通过验收。新增省级以上研发平台15家，慈湖高新区与中科院过程所共建新材料科技园。设立青年人才工作站，引进培育各类人才2.4万人、高层次科技人才团队29个。推进全国质量强市示范市创建，新增中国驰名商标2个、省名牌产品17个，国家机床产品质量监督检验中心正式批准成立，博望区荣获全国知名品牌示范区称号。提升开放型经济水平。深化对欧对台合作，新增欧资企业4家、台资企业12家。积极融入长三角，建立宁马、合马常态化对接合作机制，重点合作项目顺利推进。大力发展口岸经济，完成港口货物吞吐量1.2亿吨、集装箱量18万标箱，分别居全省第2、第3；港口资源整合有序推进；马鞍山综合保税区、肉类进口指定口岸正式运行。

****（六）加大统筹力度，着力推进协调发展。****提升主城区功能品质。马濮路、红旗南路贯通建成通车，新建公园绿地8处、公共绿地196公顷；“两治三改”深入实施，2.12万平方米违法建设完成拆除，143.28亩违法用地得到治理，57个棚户区、城中村、老旧小区完成改造。增强载体经济实力。三县GDP占全市39.3%，当涂县位列全国百强县第69位；三区发展态势良好，主要指标增速较快；三个国家级园区落地亿元以上工业项目79个，占全市40.7%。经济强镇建设成效显著，财政收入亿元以上镇达20个，太白镇财政收入超10亿元。实施乡村振兴战略。现代农业加快发展，农产品加工产值增长9.5%，新增新型农业经营主体719个、“三品一标”56个，获批国家地理标志证明商标3个，湖阳镇被评为全国“一村一品”示范乡镇。农村人居环境整治三年行动扎实推进，“三大革命”深入实施，建成农村道路畅通工程314公里，建设省级美丽乡村20个。

****（七）加强社会建设，着力增进民生福祉。****加大民生保障投入。民生支出192.59亿元，占财政支出85.1%。33项民生工程、6件民生实事全部完成。在全省率先建立被征地农民养老保障待遇正常调整机制，率先实现城乡低保标准一体化。增加基础教育供给，新建改建公办幼儿园7所，推进义务教育学校标准化建设，安师大附属郑蒲港学校建成招生；职业教育、高等教育、特殊教育同步提升。加强文明创建长效管理。市志愿服务中心建成运行。国家卫生城市通过复审，健康城市加快创建，第六次荣获全国无偿献血先进市；建成养老服务设施103个，新增养老床位1089张。推进公共文化服务体系建设，新增城市悦书房12个，含山县图书馆、当涂县博物馆建成开馆。成功举办第30届李白诗歌节。同步推进群众体育和竞技体育，我市代表团在省第十四届运动会上荣登金牌榜第3。加强社会综合治理。“平安马鞍山”建设成效明显，社会治安防控体系更加完善，人民群众安全感达到97.83%。安全生产攻坚行动扎实推进，“深重促”专项行动深入开展。国家食品安全示范城市创建通过中期评估验收，粮食安全责任制全面落实。外事侨台、民族宗教、人民防空、拥军优属、防震减灾工作实现新发展，工会、共青团、妇女儿童、残疾人、红十字事业取得新进步，地方志、档案、邮政管理等工作再创新业绩。

    各位代表！

    过去一年，是本届政府的开局之年。为了开好局、起好步，我们更加注重政府自身建设。加强政府政治建设。树牢“四个意识”，做到“两个维护”，坚决贯彻落实党中央国务院、省委省政府和市委的各项重大决策部署，确保全市经济社会发展与党中央要求对标对表，步调一致。加强政府法治建设。坚持依宪施政、依法行政，制定市政府立法五年规划，首部政府规章正式出台；健全决策议事会议制度，努力做到民主决策、科学决策；自觉接受人大法律监督和政协民主监督，办理议案建议160件、政协提案316件，办复率达100%。加强政府作风建设。严格执行中央八项规定精神和省、市实施细则，严格控制“三公”经费支出、预算追加、一般性出国考察和机构编制；扎实开展“讲严立”专题警示教育。加强党风廉政建设。认真落实全面从严治党主体责任，着力加强权力运行监督和廉政风险防控，持续强化公共资金、公共资源、公共资产、公共工程等重点领域监管。

    各位代表！

    过去的一年，成绩来之不易。我们经历了多重困难和风险挑战。这些成绩是在经济下行压力加大的背景下取得的，这里既有市场出清导致部分企业退规、停产，也有政策性淘汰关闭过剩产能；这些成绩是在完成各种整改任务的背景下取得的，全年整改完成固废问题56个、中央和省环保督察信访件及反馈问题1385个、土地例行督察反馈问题246宗、国务院大督查反馈问题16个、扶贫专项巡视和省委巡视交办问题24个、“大棚房”专项整治问题11个；这些成绩是在成功防范化解一批重大风险的背景下取得的，全年处置涉军、涉教、涉企、涉房地产等突出信访问题53个，打掉涉黑涉恶团伙40个，有效防控了非洲猪瘟重大动物疫情；这些成绩是在宏观政策趋紧、严控政府隐性债务的背景下取得的，不仅要严控政府融资增量，而且要有力化解债务存量，想干事与无钱干事的矛盾十分突出。在困难和压力面前，全市上下敢于担当、勇于作为，付出了艰辛的努力。这些成绩是习近平新时代中国特色社会主义思想特色社会主义思想科学指引的结果；是省委、省政府和市委正确领导的结果，是市人大、市政协大力支持的结果；是全市人民撸起袖子加油苦干的结果。在此，我代表市人民政府，向全市人民，向市人大代表、市政协委员、离退休老同志，向市各民主党派、工商联、无党派人士、人民团体和各界人士，向中央和省驻马单位，向驻马解放军指战员、武警官兵、政法公安干警和消防救援队伍指战员，向所有关心、支持、参与马鞍山建设发展的境内外投资者、海内外朋友，表示衷心的感谢和崇高的敬意！

    稳不忘忧，好中知难。我们清醒地看到，我市经济社会发展还存在不少问题和不足：战新产业集聚度不高，转型升级步伐不够快；实体经济困难，企业亏损面扩大；财政收支矛盾突出，还本付息压力增大；污染治理基础不牢，治本任务艰巨；历史遗留问题多发，风险隐患上升；少数干部表态多调门高、行动少落实差，一些部门形式主义、官僚主义、好人主义、痕迹主义不同程度存在；一些领域腐败现象时有发生。我们要直面问题，抓住主要矛盾，采取得力措施，切实加以解决。

****二、2019年工作思路目标****

    进入新时代，市委九届七次全会描绘了"1234"美好蓝图，这是马鞍山人民共同的梦想，我们都是追梦人。过去的一年，我们为实现这一梦想努力奔跑，新的一年我们要接续奋斗，久久为功，逐梦前行。

    展望新的一年，我们有挑战，也有机遇。从挑战的一面来看，我们面临着稳中有变、变中有忧的外部环境，自身也面临着一些新的课题。比如，一些重点企业生产有可能冲高回落，房地产市场难以高位运行，一些历史遗留问题有可能集中暴露，等等，这些都为做好2019年工作增加了变数和困难，必须做好沉着应对的准备。但辩证分析，我们也面临许多难得发展机遇和有利条件。比如，新中国成立70周年、改革开放再出发的重大政治机遇；深入推进长三角一体化发展、长江经济带发展的国家战略机遇；继续实施积极的财政政策和稳健的货币政策逆周期调节的宏观政策机遇，等等。从长期大势认识当前形势，从全局看局部，从未来看当下，我们仍然处于应当大有作为也能够大有作为的重要战略机遇期。我们要增强信心，保持定力，抓住机遇，用好机遇，扎扎实实做好马鞍山自己的事。

    今年政府工作的总体要求是：坚持以习近平新时代中国特色社会主义思想特色社会主义思想为指导，全面贯彻党的十九大和十九届二中、三中全会精神，认真贯彻落实中央、省委经济工作会议部署，按照市委九届七次、八次全会要求，统筹推进“五位一体”总体布局，协调推进“四个全面”战略布局，统筹抓好稳增长、促改革、调结构、惠民生、防风险、保稳定各项工作，以优异成绩庆祝中华人民共和国成立70周年。

    综合考虑，今年全市经济社会发展的主要预期目标为：地区生产总值增长8%-8.5%，财政收入增长8.5%，固定资产投资增长10%，社会消费品零售总额增长10.5%，进出口增长高于全省平均水平，城镇居民人均可支配收入增长与经济增长同步，农村居民人均可支配收入增长9%，城镇登记失业率控制在4%以内，单位GDP能耗、主要污染物排放总量完成省下达指标。

    做好今年政府工作，必须着重把握好以下几点：

****一是坚持稳中求进。****“稳”是发展的前提和基础。面对复杂多变的宏观环境，我们必须坚持“稳”字当头，全面落实稳就业、稳金融、稳外贸、稳外资、稳投资、稳预期“六稳”工作部署，以稳中求进应对稳中有变。但“稳”并不意味着一味求稳，必须保持永不懈怠的精神状态和一往无前的奋斗姿态，能快则快，瞄准合肥、芜湖等标兵，奋力追赶。

****二是坚持转型升级。****转型升级是推动马鞍山高质量发展的主题主线，也是深化供给侧结构性改革的必由之路。要围绕生态福地建设，统筹推进森林城市、健康城市、智慧城市建设，加快城市转型升级步伐；要围绕智造名城建设，着力推进智能装备制造（机器人）、轨道交通装备、高端数控机床三大战新基地建设，努力在产业转型升级上取得新成效。

****三是坚持扩大开放。****马鞍山是资源型城市，在转型过程中，必须用好国内国外两个市场、两种资源。我们要让开放的大门越开越大，持续拓展对欧对台合作；深度对接长三角、珠三角；加快融入南京都市圈、合肥都市圈，构建高水平对外开放平台、载体和通道，不断提高开放型经济发展水平。

****四是坚持一体化发展。****长三角一体化和长江经济带发展为马鞍山提供了千载难逢的发展机遇，我们要敢于和高手过招、同台竞技。对外要拓宽长三角视野，强化一体化 意识，更快速度推进重大基础设施互联互通，更大力度承接产业资本转移，更高水平实现基本公共服务合作共享。对内要统筹推进一江两岸、新老城区以及城乡协调发展，做大园区经济和县域经济规模。

****五是坚持以人民为中心。****人民对美好生活的向往，就是我们的奋斗目标。我们不仅要多措并举，稳步增加城乡居民收入，改善和保障民生，抓好脱贫攻坚和困难群众帮扶，力促民富；而且要切实抓好公共安全、信访维稳、社会治理、扫黑除恶等社会稳定工作，力保民安。

****三、2019年主要工作任务****

    今年经济社会发展任务十分繁重，我们要着重抓好以下九个方面工作：

****（一）打好三大攻坚战关键之役。****突出抓重点补短板强弱项，打好重点战役，确保再战再捷。

    加大风险防控力度。持续整治互联网金融，严厉打击逃废金融债务等违法违规行为。有序化解政府隐性债务，继续实施债务规模和融资成本“双控”措施。强化对融资平台、产业投资基金、PPP项目和政府购买服务的规范化管理，做到坚定、可控、有序、适度，确保马鞍山成为金融稳健安全城市。同时要抢抓机遇，积极争取国家专项债券。加快市农商行、马钢嘉华上市进程，新增上市挂牌企业50家，直接融资力争达到150亿元以上。

    推进精准脱贫攻坚。狠抓各项扶贫政策落实，确保所有未脱贫户全部脱贫。深入开展“百企帮百村”精准帮扶行动，完善“四带一自”产业扶贫、“三有一网”点位扶贫模式，推动“金菜地”“绿游游”“金稻渔”等特色产业扶贫工程提质增效。研究解决收入水平略高于建档立卡贫困户的群体缺乏政策支持等新问题，及早谋划脱贫攻坚目标完成后的工作思路，统筹开展无扶贫开发任务“一县三区”农村困难群众帮扶工作。脱贫攻坚工作要继续走在全省前列。

    加强生态环境保护。这两年我们虽然在生态环保上做了大量工作，取得了显著成效，但生态环境问题依然十分严重。我们必须坚持长短结合、标本兼治、城乡统筹、内外联控的方略，毫不松懈地保持高压态势，坚守阵地巩固成果。要深入实施蓝天保卫战三年行动计划，确保PM2.5平均浓度持续下降、空气优良天数同比上升。全面实施水污染防治行动计划，严格落实河长制、湖长制，长江马鞍山段国控断面水质优良率继续保持100%；加快国家黑臭水体治理示范城市建设，提升中心城区水环境治理水平，到2020年彻底消除主城区黑臭水体。全面实施土壤污染防治行动计划，推行生活垃圾分类处理，严厉打击固体废物非法转移、倾倒、处置。加快推进生态环保重点工程建设，一般工业固废集中处置和循环利用中心建成运营，得胜河水环境综合治理全面完工。加快建设美丽长江（马鞍山）经济带，谋划实施长江马鞍山东岸及采石河沿线生态环境综合治理。

****（二）坚定不移推动经济高质量发展。****坚持以供给侧结构性改革为主线，全面贯彻“巩固、增强、提升、畅通”八字方针，着力提高经济发展质量和效益。

    加快智造强市建设。雨山区、市经开区、博望区要聚力三大战新基地建设，其他载体应进一步明确产业定位，提高集中度，大力发展电子信息、新能源、生物医药、新材料、绿色食品等产业，全市战新产业产值增长15%以上。开展机器换人“十百千”工程，引导企业“设备换芯”“生产换线”，新增一批省级以上数字化生产车间和智能工厂。实施亿元以上重点技改项目70个，完成投资增长9%。企业兼并重组30家。推动先进制造业和现代服务业深度融合，支持发展互联网、大数据、人工智能、数字经济等新兴产业。引进国内知名企业，合作建设大数据中心、云计算中心、信息安全中心、全息数字马鞍山，加快智慧城市建设。实施“企业登云”计划，推动80家以上企业“上云”，两化融合管理体系贯标企业突破17家。加强质量强市建设，持续实施质量提升行动。积极争取省级军民融合深度发展试点，培育军民融合优势产业集群。

    持续加大项目投入。项目是经济工作的主抓手，今天的项目就是明天的增长点，今天的投资结构就是明天的产业结构。今年我们将继续开展“项目建设年”和“双集中”活动，实施重点项目630个，完成投资750亿元，突出抓好市级调度100个项目，确保易锐集成光电子产业基地、音飞智能储存设备等150个项目开工建设，永途新能源汽车、中铁轨道交通装备等78个项目竣工投产。瞄准智能制造、生态环保、基础设施等关键领域，谋划一批重点项目，全市重大项目库规模达2000亿元，争取更多项目纳入国家和省盘子。加强项目用地、融资、能耗指标、市政配套等方面的统筹。坚持招大引强，继续实行市领导领衔重大招商项目制度，引进20亿元以上项目10个以上，全年实际利用省外亿元以上项目资金增长8%，利用外商直接投资增长7%。

    激发居民消费潜力。落实个人所得税专项附加扣除政策，千方百计增加居民收入，探索低收入群体基本生活现金救助，提高居民消费能力。培育新的消费产品和业态，推动传统商贸业转型发展。鼓励社会力量参与健康、养老、家政等服务供给，促进消费提档升级。加快文旅融合，凌家滩国家考古遗址公园挂牌运营，采石5A级旅游景区景观质量等级通过国家级评定。深入推进“电商马鞍山”建设，华东农产品物流园竣工投产。改善消费环境，严厉打击制售假冒伪劣商品违法行为，开展“放心消费”创建，让老百姓安心消费、舒心消费。

****（三）持续深入实施创新驱动战略。****以国家创新型城市建设为抓手，深入实施大众创业、万众创新，培育更多“四新经济”。

    加强创新能力建设。加快创新主体培育，新认定高新技术企业50家、培育科技“小巨人”企业50家。促进产学研用深度融合，鼓励企业对接高校科研院所，支持共性技术创新平台建设，省级以上研发机构突破150家，规上工业企业研发机构覆盖率达到35%以上。省级以上科技企业孵化器达到10家，在孵企业（团队）超过600家。高新技术产业增加值增长15%以上，全社会研发经费投入占GDP比重达2.65%，每万人发明专利突破20件。

    促进创新人才集聚。坚定不移走“人才兴市”之路，大力实施“诗城英才”计划，继续推进人才引进“龙马”“骏马”“驿马”工程、本土人才培育"1221"工程，着力集聚各方面优秀人才。加快“技工大市、职教名城”建设，优化职教资源和院校布局，促进校企融合发展，新增各类技能人才1.88万人。优化人才发展环境，完善人才服务保障体系，为各层次人才提供安居、医疗、配偶就业、子女入学、出入境审批等方面的便捷服务。

    推动园区高质量发展。园区是全市经济发展的引擎。市经开区、慈湖高新区、郑蒲港新区要招大引强、扶优扶强、做精主业、做优服务，在全国全省开发区排名中争先进位，提高对全市经济发展的贡献度。统筹推进6个省级开发区建设，推动当涂经开区争创国家级。创新开发区管理体制，加大市级权限下放力度。在国家级开发区探索设立行政审批局，实行“一枚印章管审批”。加快郑蒲港新区法定机构改革试点。支持宿马产业园加快发展。

****（四）大力推动新一轮改革开放。****立足新起点，改革再出发，开放不停步，增强经济发展活力和内生动力。

    深化重点领域改革。完成市县政府机构改革。持续推进“放管服”改革，巩固扩大“证照分离”、相对集中行政许可权、“减证便民”改革成果；提升“互联网+政务服务”水平，抓好“企业开办全程网上办”改革试点。坚持市场化导向，推动江东控股及县区城投公司转型发展；推进钢城深度融合，支持马钢多元化发展、国际化经营，加快培育千亿级支柱企业。深化财税金融改革，注重培育地方税体系。深化医药卫生体制改革，完善医联体、医共体、专科联盟内部运行机制，加快建立现代医院管理制度。

    促进民营经济大发展。全年力争民营经济增加值突破1000亿元大关。大幅度降低土地使用税。设立5亿元支持民营企业发展资金，引导各载体设立民营企业纾困救助基金。增加小微企业续贷过桥资金规模和周转次数；加大对民营中小企业担保贷款力度；支持金融机构完善信贷激励机制，加大信贷投放。树立尊重企业尊重企业家良好风尚，规范文明执法检查，坚决杜绝政策执行简单化和“一刀切”。常态化推进“四送一服”双千工程，开展民营企业大走访和“一企一组一策”，全面对接南京，创优营商环境。

    提升对外开放水平。加强对欧对台经贸合作，拓宽对欧合作通道，加快建设中欧产业合作园，引进欧资项目不少于10个；加快筹建台湾半导体产业园，引进台资项目不少于20个。加大承接长三角、珠三角产业转移力度，实现产业协同错位发展。加快综保区建设，积极参与安徽自贸区创建。对接融入“一带一路”，调整优化外贸结构，加快发展跨境电子商务，培育引进龙头企业，支持马钢、十七冶等重点企业走出去，开拓多元国际市场，保持外资外经外贸稳定增长。

****（五）全面融入长三角一体化发展。****坚持把马鞍山发展放到长三角城市群中审视谋划，发挥优势，找准定位，全方位对接融入。

    加强战略研究和谋划。积极参与长三角一体化发展规划编制和省实施方案的制定，着手制定宁马一体化发展和一江两岸协调发展规划，着力构建定位清晰、特色鲜明的区域协调发展格局。加快编制各类专项规划，做好对接衔接，为全面融入长三角更高质量一体化发展奠定坚实基础。

    加快基础设施互联互通。落实南京都市圈系列合作框架协议。推动省际、城际协调，确保巢马城际铁路今年开工建设，宁马城际铁路、宁和城际铁路二期纳入长三角区域城际铁路网规划，郑蒲港铁路、商合杭高铁含山段主体建成，深化马鞍山-南京-镇江城际铁路、宁宣黄铁路前期工作。推动宁马高速改造开工建设，加快打通城际断头路，推进S328与南京新浦合公路、G347与苏S356对接。推动郑蒲港与合肥港深度合作。

    开展全方位对接合作。毗邻地区要率先融合发展，推动和县乌江新区深度融入南京江北新区，集聚发展电子信息、智能装备制造等新兴产业；推动含山县与巢湖市、巢湖经开区深入合作，共建环巢湖全域旅游示范区；支持马鞍山软件园与南京软件谷开展战略合作，推动博望区与南京江宁区、溧水区深度融合发展，加快慈湖高新区宁马青年创业谷等合作平台建设。推进污染联防联治，共建生态走廊和生态屏障。深化科技、教育、医疗等社会公共服务合作。

****（六）着力提升城市功能品质。****坚持“组团式、网格化、田园城市”发展思路，推动城市双修，进一步提升城市颜值。

    优化城市布局。坚持规划引领，加快新一轮城市空间总体规划修编。完成城市总体设计报批，开展重点地块城市设计编制。建成空间规划平台，建立多规合一管理信息互通机制，实现一本规划管控。深入实施主体功能区规划，全面提升国土空间开发保护质量和效率。

    提升城市功能。“四合一”计划完成投资300亿元。支持秀山新区加快发展。加快银杏大道东延等20条城市道路建设，推动背街小巷、园区和小区道路改造提升。市体育会展中心、市气象防灾减灾指挥中心、综合客运中心等公益性项目建成使用。继续推进“两治三改”，集中开展城市拆违攻坚行动。完成棚户区、城中村改造2814户，改造老旧小区19个；试点推进多层住宅电梯加装工作。开展“优先发展公共交通示范城市”创建。依法依规推进征迁拆迁，完成省政府下达闲置土地清理处置任务。做好第三次国土调查、第四次经济普查工作。严格落实林长制，创建成为国家森林城市，新增造林1.8万亩、四旁植树1.5万亩，新建或改造公园绿地6处、湿地公园4个。

    强化城市治理。完善文明创建长效管理机制，提升市民文明素养，打造全国文明城市升级版。实施城市道路、游园、广场、高速出入口“六化”提升工程，加大城市公厕、垃圾中转站改造力度。持续推进烟花爆竹禁放、犬只文明饲养，着力解决占道经营、乱停乱放、小区乱象等突出问题。严格执法标准，规范城市管理执法行为。

****（七）深入实施乡村振兴战略****。坚持农业农村优先发展总方针，按照“二十个字”总要求，努力打造乡村振兴马鞍山样板。

   推进农业现代化。开展产业兴村强镇、加工强县创建、产业化龙头企业提质、品牌农业创建等系列行动，农产品加工产值增长8%。新增各类新型经营主体500个、农业产业化龙头企业10个。实施休闲农业和乡村旅游提升工程，办好农民丰收节以及和县蔬菜博览会、当涂桃花节等特色文旅活动。支持含山县、当涂县争创国家级农产品质量安全示范县。坚决守住“农地姓农”底线，深入开展殡葬设施违规建设专项整治，新增高标准农田7万亩。

    改善农村人居环境。完善村庄布点规划，推进中心村建设。继续实施农村人居环境整治“三年行动”，打好农村污染治理攻坚战，一体化推进农村环境“三大革命”，着力改善村容村貌、户容户貌。加强农村基础设施建设，建成“四好农村路”扩面延伸工程468.6公里，支持和县争创全国“四好农村路示范县”。健全自治、法治、德治相结合的乡村治理体系，实施村民道德建设工程，培育淳朴民风、文明乡风、良好家风。

    持续深化农村改革。实施农村集体经济壮大“三年行动”，全面完成农村集体产权制度和“三变”改革。创新村级集体经济管理和运营机制，成立股份经济合作社180个，完善法人治理结构，规范收益分配，促进集体经济发展和农民稳定增收。推进乡村企业家人才队伍建设“百千万”工程，实施农民工职业技能提升计划，鼓励社会各界投身乡村振兴。

    做大做强县域经济。推动含山县高端铸造、和县现代化工、当涂县智能家电等产业集群发展，培育县域特色产业集群基地，提升县域经济竞争力。高标准推进县城建设，增强县域中心功能。加快新型城镇化步伐，支持黄池、香泉、运漕等国家和省级特色小镇创新发展，推动向山、博望等中心镇加快发展。

****（八）尽心尽力保障改善民生。****坚持以人民为中心的发展思想，着力补齐民生短板，优化公共服务供给。

    稳定和扩大就业。实施就业优先政策，新增城镇就业4.5万人。完善重点群体就业援助制度，实施高校毕业生就业创业促进计划，加大失业人员就业帮扶力度，抓好军转干部和退役士兵就业创业。提升青年创业园、大学生创业园、农民工返乡创业示范园服务功能。开展援企稳岗补贴工作，鼓励企业稳定现有就业岗位。

    促进教育文化发展。深化教育综合改革，加快学前教育普惠发展、义务教育优质均衡发展，新建、改扩建公办幼儿园7所，新增办学集团3个，加快红星中学新校区、秀山中学建设，建成博望高中并投入使用；扩大职业教育规模，支持民办教育规范发展。培育和践行社会主义核心价值观，支持当涂县新时代文明实践中心建设试点工作。推进基层文化服务效能提升，通过国家公共文化示范区复审验收。高质量办好李白诗歌节、“江南之花”等群众文化活动。积极培育志愿服务文化。

    提供优质公共服务。科学谋划和安排年度民生工程、为民办实事项目。推进全民参保计划，开展扩面普查专项行动。完善社会保障体系，帮助城市困难职工脱困解困，实施残疾人服务关爱工程。完成养老服务体系建设三年行动计划，开展居家和社区养老服务改革试点，江东颐养中心建成运营。加快健康马鞍山建设，力争跻身全国健康城市示范市行列，打造一批省级健康特色小镇，支持含山县、和县创建国家卫生县城。适应实施全面两孩政策，创新计划生育服务工作。推进体育强市建设，广泛开展全民健身活动，办好市十二运会。加强外事侨台、档案、地方志、供销等工作，积极发展工会、妇女儿童、青少年、科普、关心下一代等事业。

****（九）切实维护社会大局稳定。****落实稳定第一责任，构建共建共治共享体系，着力形成维护社会稳定的长效机制。

    创新社会治理方式。加强社区建设，严格社区服务事项准入，切实减轻社区负担。完善社区综合服务设施，提升社区网格管理精细化水平。健全公共法律服务体系，加大法律援助和全民普法力度。借鉴“枫桥经验”，推广社会矛盾纠纷多元化解机制，开展县乡村“联建、联创、联评”活动，创新信访工作方式，全力化解信访积案。发挥"12345"市长热线等平台作用，畅通群众诉求渠道，及时解决群众身边的烦心事。巩固民族团结、宗教和睦局面。

    提升应急管理能力。推进安全生产“铸安”行动常态化实效化，持续加强风险管控“六项机制”建设，突出抓好事故隐患排查治理和重点行业领域专项整治，坚决防范各类生产安全事故。积极推进国家安全发展示范城市创建。完善应急预案体系，加强应急演练和救援力量建设，提升综合防灾减灾救灾能力。加强气象、地震、红十字等工作。

    巩固提升公共安全。深化“平安马鞍山”创建，加强社会治安防控体系和能力建设，全面启动“雪亮”工程。深入推进扫黑除恶专项斗争，严厉打击各类违法犯罪行为。通过国家食品安全示范城市创建验收。加强非洲猪瘟等重大动物疫情防控，保持畜禽产品市场供应和价格稳定。落实粮食安全责任制。推进国防教育、国防动员、人民防空建设，实现全国双拥模范城“四连冠”。

****四、全面加强政府自身建设****

    新的征程已经开启。新征程要有新气象，更要有新作为。我们将牢记职责使命，全面加强政府自身建设，全面提高政府履职水平。

****（一）加强政治建设，练就过硬本领。****树牢“四个意识”，坚定“四个自信”，坚决做到“两个维护”，创造性贯彻落实党中央国务院、省委省政府重大决策部署，坚决服从市委领导，坚定执行市委决定。把建设学习型政府摆上突出位置，增进专业知识、提高专业素养、培养专业能力。加强调查研究，在实践中找思路想办法，努力成为解决实际问题的能手。增强互联网思维，善用信息化手段，辅助决策施政，提高行政效能，走好网上群众路线。

****（二）严格依法办事，提升治理水平。****自觉遵守宪法和法律，把政府工作全面纳入法治轨道。严格执行民主集中制，推进重大事项合法性审查全覆盖，更加重视发挥法律顾问作用，做到依法科学民主决策。加强政府立法，规范公正文明执法，加快统一执法和监督平台建设。自觉接受市人大及其常委会法律监督、工作监督和市政协民主监督，高质量办理人大代表议案建议和政协委员提案，广泛听取各民主党派、工商联、无党派人士和人民团体意见。

****（三）勇于担当作为，践行为民宗旨。****以勇于担当的政治品格，把该担的责任担起来、该抓的工作抓到位，做到在其位、谋其政、干其事、求其效，坚决防止“为官不为”。以只争朝夕、时不我待的精神，跟上新时代，抢抓新机遇，抓紧每一天，干好每件事，坚决克服慢动作、跟不上现象。以求真务实的作风，说老实话、办老实事、做老实人，防止空喊政治口号的形式主义，防止简单粗暴的命令主义。摒弃“痕迹管理”，切实减轻基层负担。

****（四）从严正风肃纪，加强廉政建设。****认真落实全面从严治党主体责任和“一岗双责”，强力推进省委巡视反馈问题整改，扎实推进政府系统党风廉政建设和反腐败工作。支持纪检监察机关依规依纪依法履行职责，强化审计监督，坚决惩治各类腐败行为。政府工作人员要廉洁修身、勤勉尽责，干干净净为人民做事，绝不辜负人民公仆的称号。

    各位代表！奋斗托起梦想，拼搏成就辉煌。让我们紧密团结在以习近平同志为核心的党中央周围，在省委、省政府和市委的坚强领导下，锐意进取、埋头苦干，全面开创新时代马鞍山高质量发展新局面，以优异的成绩迎接新中国成立70周年！
